# Cross‐national harmonization of cognitive impairment and dementia diagnosis across US and Latin American populations

**DOI:** 10.1002/alz.086959

**Published:** 2025-01-09

**Authors:** Jorge J. Llibre‐Guerra, Jordan Weiss, Jing Li, Chris Soria, Ana M Rodriguez‐Salgado, Juan J. Llibre‐Rodriguez, Ivonne Z. Jiménez‐Velazquez, Daisy M Acosta, Mao‐Mei Liu, William H Dow

**Affiliations:** ^1^ Washington University School of Medicine, St. Louis, MO USA; ^2^ Stanford University, Stanford, CA USA; ^3^ CHOICE Institute, University of Washington, Seattle, CA USA; ^4^ Department of Demography, University of California at Berkeley, Berkeley, CA USA; ^5^ Global Brain Health Institute, San Francisco, CA USA; ^6^ Dementia Research Unit/Medical University of Havana, Havana, Havana Cuba; ^7^ University of Puerto Rico, School of Medicine, San Juan Puerto Rico; ^8^ Universidad Nacional Pedro Henriquez Ureña, Santo Domingo, Distrito Nacional Dominican Republic

## Abstract

**Background:**

Cross‐national comparisons of dementia prevalence are essential for identifying unique determinants and cultural‐specific risk factors, but methodological differences in dementia ascertainment across countries hinder global comparisons. This study maps the 10/66 Dementia Research Group algorithm for dementia ascertainment, widely used and validated in lower‐ and middle‐income countries, to the U.S.‐based Aging, Demographics, and Memory Study (ADAMS), and validates it for use in the U.S.

**Methods:**

We conducted pre‐statistical harmonization across the ADAMS and the 10/66 studies in Latin America to identify common measures, which were then utilized to develop a modified 10/66 algorithm. We computed dementia probabilities using the modified 10/66 algorithm and the optimal threshold for dementia ascertainment was established by maximizing sensitivity and specificity against ADAMS' clinical gold standard diagnosis. We then compared this algorithm to a TICS‐based algorithm developed in ADAMS and commonly used in the U.S. Health and Retirement Survey. Further analysis included an examination of the age‐adjusted educational gradients in predicted dementia prevalence, allowing for a comprehensive assessment of the modified algorithm’s validity and its comparative performance.

**Results:**

The sample comprised 5,649 adults ages 70 and over (539 from ADAMS and 5,109 from 10/66 studies).10/66 participants had a lower education level (60% had primary school or less) relative to the ADAMS cohort (69.8% possessed at least a high school education). The modified 10/66 diagnostic algorithm performed similarly in the 10/66 data as compared to the original 10/66 algorithm. In the ADAMS data, the modified 10/66 algorithm outperformed the TICS algorithm across several metrics: it demonstrated higher sensitivity (90% vs 77%), specificity (90% vs 74%), accuracy (90% vs 75%), and Area Under the Curve (AUC) (90% vs 76%). The modest ADAMS sample size precluded statistically precise comparisons by education, but education gradients in predicted dementia were broadly similar across algorithms.

**Conclusions:**

The modified 10/66 algorithm effectively classifies dementia in US studies and is suitable for cross‐national comparisons, supporting its use for international studies. This facilitates future harmonization efforts of dementia diagnostic tools and enhances cross‐country dementia research.

